# Supplementary figures and images for: Functional Characterization of the N-Terminal Disordered Region of the piggyBac Transposase
Source: Int J Mol Sci. 2022 Sep 7;23(18):10317. doi: 10.3390/ijms231810317 (PMC9499001; doi:10.3390/ijms231810317)

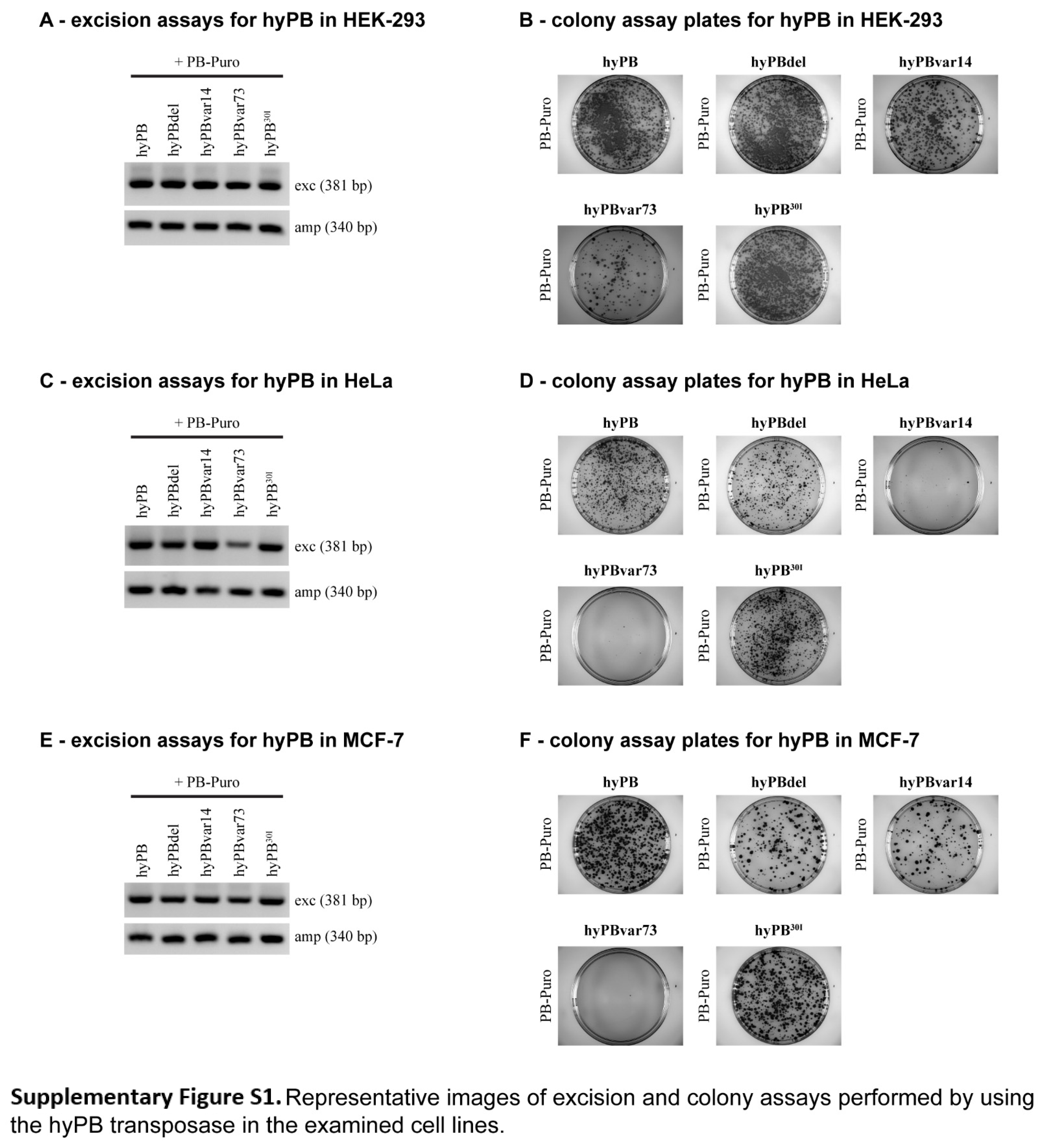

Supplement: Supplementary file 1 [file ijms-23-10317-s001.zip › Suppl_Figure S1.tif]

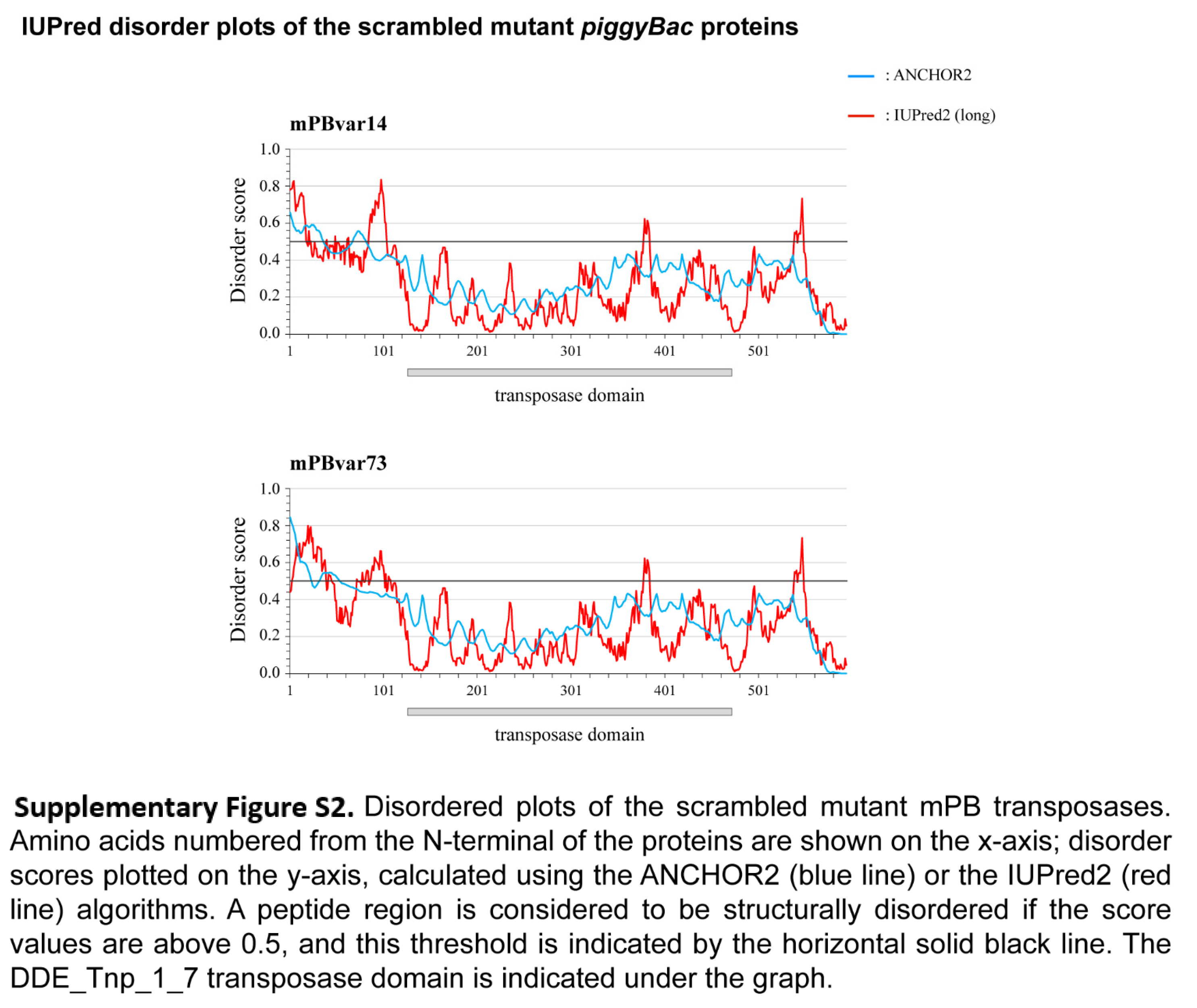

Supplement: Supplementary file 1 [file ijms-23-10317-s001.zip › Suppl_Figure S2.tif]
